# Leave Against Medical Advice among Neonates Admitted to Neonatal Intensive Care Unit in a Tertiary Care Centre: A Descriptive Cross-sectional Study

**DOI:** 10.31729/jnma.8212

**Published:** 2023-07-30

**Authors:** Yograj Sharma, Om Krishna Pathak, Asmita Sharma, Suvekhsya Khanal, Niruta Baral, Sagar Shrestha, Pradeep Adhikari

**Affiliations:** 1 Department of Paediatrics, Bharatpur Hospital, Bharatpur, Chitwan, Nepal; 2 Bharatpur Hospital Nursing Program, Bharatpur Hospital, Bharatpur, Chitwan, Nepal; 3 Department of Anaesthesiology, Bharatpur Hospital, Bharatpur, Chitwan, Nepal

**Keywords:** *leave against medical advice*, *neonatal intensive care unit*, *neonates*, *prevalence*

## Abstract

**Introduction:**

Leave against medical advice is a condition in which a patient leaves the hospital against the treating physician's recommendation and is a sensitive issue occurring frequently in neonatal intensive care units across the developing world. Investigating the causes of newborn deaths is essential as there is high neonatal mortality rate in context of Nepal and a huge gap between that rate and desired outcomes. Self-discharge of sick neonates from hospitals further increases the chance of death. Hence, this study aimed to find out the prevalence of leave against medical advice among neonates admitted to the neonatal intensive care unit in a tertiary care centre.

**Method:**

This descriptive cross-sectional study was conducted among patients admitted to the Neonatal Intensive Care Unit from 14 April 2021 to 13 April 2023 after obtaining ethical approval from the Institutional Review Committee (Reference number: 077/78-021). The patient's demographic and clinical characteristics and reasons for self-discharge were recorded. Convenience sampling was done. Point estimate and 95% Confidence Interval were calculated.

**Results:**

Among 1352 neonates, the prevalence of leave against medical advice was 119 (8.80%) (7.29-10.31, 95% Confidence Interval).

**Conclusions:**

The prevalence of leave against medical advice from the neonatal intensive care unit was lower than in other studies done in similar settings.

## INTRODUCTION

Leave against medical advice (LAMA) is a condition in which a patient (the parents or caregivers in the case of a neonate) chooses to leave the hospital before the treating physician recommends discharge.^1^ It is also called discharge against medical advice (DAMA), self-discharge or leave before their visit (or admission) is complete (LBVC).^1,2^

Leaving the hospital against advice not only raises clinical, legal and ethical issues for the treating physician but also exposes the patient to the risk of an inadequately treated medical problem resulting in readmission and other significant problems including death.^3,4^ Limited studies in pediatric populations from developing countries indicate that it is more prevalent among neonates and has serious consequences owing to their limited physiological reserves.^5,6^

This study aimed to find out the prevalence of LAMA in the neonatal intensive care unit of a tertiary care centre.

## METHODS

This descriptive cross-sectional study was conducted in the Neonatal Intensive Care Unit of Bharatpur Hospital, Bharatpur, Chitwan, Nepal from 14 April 2021 to 13 April 2023 for a period of two years where data collection was started after receiving ethical approval from the Institutional Review Committee of Bharatpur Hospital (Reference number: 077/78-021). After informed consent from the parents or immediate caretaker, all neonates admitted to the NICU were enrolled in the study. Newborn babies whose parents denied consent were excluded. Convenience sampling was used. The sample size was calculated by using the following formula:


$$\begin{array}{ll} \text{n} & ={\text{Z}}^{2}\times \frac{\text{p}\times \text{q}}{{\text{e}}^{\text{2}}}\\ & =1.96^{2}\times \frac{0.18\times 0.82}{0.03^{2}}\\ & =630 \end{array}$$

Where,

n = minimum required sample size
Z = 1.96 at 95% Confidence Interval (CI)
p= prevalence of LAMA discharge in NICU from a previous study, 18%^7^
q= 1-p
e= margin of error, 3%

The calculated sample size is 630. On doubling the sample size, it becomes 1260. However, a sample size of 1352 was included in the study.

Patient particulars including name, age, gender, address, religion etc. and clinical & obstetric parameters like maternal age, gestational age, birth weight, place and mode of delivery etc. were recorded at admission to the NICU. The categorization of ethnicity according to caste was done as outlined in the Population Situation Analysis of Nepal (UNFPA Nepal 2017).^8^ Morbidities encountered during treatment in the NICU were followed and recorded.

Clinical diagnoses were made based on the standard guidelines being used in the unit. Relevant laboratory investigations and imaging techniques were used whenever necessary. Sepsis was diagnosed on clinical grounds along with c-reactive protein (CRP), complete blood count (CBC), blood cultures, and cerebrospinal fluid (CSF) examination. Respiratory distress syndrome (RDS), meconium aspiration syndrome (MAS) and pneumonia were diagnosed by X-ray and ultrasound of the chest along with relevant laboratory and clinical findings. Congenital heart diseases were confirmed by echocardiography.

In the case of a request for leave against medical advice (LAMA), the most senior nurse on the shift, on duty doctor and the attending consultant were informed and called in to discuss with and counsel the parents/caregivers for the need for continued hospitalization and treatment. When they insisted on discharge despite adequate counselling, they were made to sign the LAMA document and sent home. Regarding the cause of LAMA, the parents/caregivers were asked to fill out a semi-structured questionnaire prepared in the Nepali language after informed consent at the time of discharge by the principal investigator.

Data were entered in Microsoft Excel 2010 and analysed using IBM SPSS Statistics version 20.0. Point estimate and 95% CI were calculated.

## RESULTS

Among 1352 total admissions in the NICU, the prevalence of LAMA was found to be 119 (8.80%) (7.29-10.31, 95% CI). Out of a total of 119 patients leaving against medical advice, 70 (58.88%) were male with a male: female ratio of 1.4:1 (Table 1).

**Table 1:** Demographic profile (n= 119).

Variables	n (%)
**Gender**	
Male	70 (58.82)
Female	49 (41.17)
**Residence**	
Rural	65 (54.62)
Urban	54 (45.37)
**Ethnicity**	
Hill janajati	58 (48.73)
Brahmin	25 (21.00)
Hill dalit	15 (12.60)
Terai janajati	9 (7.56)
Chhetri	8 (6.72)
Muslim	3 (2.52)
Terai middle castes	1 (0.84)
**Religion**	
Hindu	90 (75.63)
Christian	17 (14.28)
Buddhist	9 (7.56)
Muslim	3 (2.52)

Seventy-two (60.50%) of the babies who took LAMA discharge were born preterm. More than three-fourths of babies were born via normal vaginal delivery 79 (66.38%) and born in a hospital setting 94 (78.99%) (Table 2).

**Table 2:** Clinical and obstetric characteristics (n = 119).

Variables	n (%)
**Gestational age (weeks)**	
<28	10 (8.40)
28 to <32	23 (19.32)
32 to <34	21 (17.64)
34 to <37	18 (15.12)
≥37	47 (39.49)
**Birth weight (grams)**	
<1000	19 (15.96)
1000 to <1500	18 (15.12)
1500 to <2500	38 (31.93)
2500 to <4000	42 (35.29)
≥4000	2 (1.68)
**Age at admission**	
<24 hours	105 (88.23)
1-7 days	10 (8.40)
>7 days	4 (3.36)
**Mode of delivery**	
Normal vaginal delivery	79 (66.38)
Caesarean section	37 (31.09)
Assisted vaginal delivery	3 (2.52)
**Place of delivery**	
Hospital	94 (78.99)
Home	15 (12.60)
Ambulance (on the way to the hospital)	5 (4.20)
Health post/clinic	5 (4.20)
**In/outborn**	
Inborn	82 (68.90)
Outborn	37 (31.09)
**Referred from other centres**	
Yes	25 (21.00)
No	94 (78.99)

The duration of hospitalization before taking LAMA was between 2-7 days in 60 (50.42%) newborns (Table 3).

**Table 3:** Duration of hospital stay before LAMA (n= 119).

Duration	n (%)
≤1 day	29 (24.36)
2-7 days	60 (50.42)
8-14 days	17 (14.28)
>14 days	13 (10.92)

Prematurity was seen in 72(60.50%), sepsis in 62(52.10%) and birth asphyxia in 47 (39.49%) newborn(Table 4).

**Table 4:** ^*^Morbidity profile of newborns with LAMA discharge (n = 119).

Diagnosis	n (%)
Prematurity	72 (60.50)
RDS	42 (35.29)
Apnea of prematurity	10 (8.40)
Pneumonia	13 (10.92)
MAS	16 (13.44)
Pneumothorax	3 (2.52)
Need of invasive mechanical ventilation	33 (27.73)
Sepsis	62 (52.10)
Birth asphyxia	47 (39.49)
Shock	19 (15.96)
Acute kidney injury	11 (9.24)
Hypoglycemia	8 (6.72)
Neonatal jaundice	17 (14.28)
Meningitis	3 (2.52)
Intraventricular haemorrhage	7 (5.88)
Seizures	10 (8.40)
Necrotizing enterocolitis	5 (4.20)
Birth defects	
Acayanotic congenital heart disease (CHD)	6 (5.04)
Complex CHD	1 (0.84)
Cleft lip and palate	3 (2.52)
Ambiguous genitalia	1 (0.84)
Ileal/duodenal atresia	2 (1.68)
Spina bifida	1 (0.84)
Congenital diaphragmatic hernia	1 (0.84)
Holoprosencephaly	2 (1.68)
Other minor defects	6 (5.04)

* **Note:** Some patients can have more than one diagnosis.

The most common reason for LAMA was poor prognosis explained by the treating physician 48 (40.33%) followed by no improvement (Table 5).

**Table 5:** ^†^Reasons for LAMA (n= 119).

Reasons	n (%)
**Financial**	
Lack of funds/unaffordability	32 (26.89)
**Social**	
Other children to take care at home	6 (5.04)
Long-distance	14 (11.76)
No one to take care of the farming/agriculture at home	24 (20.16)
Forced to take LAMA by grandparents/relatives	14 (11.76)
**Cultural**	
Religious belief on other modes of treatment	1 (0.84)
Cultural practices of other forms of treatment	2 (1.68)
**Clinical**	
Perceived improvement	13 (10.92)
No improvement	40 (33.61)
Poor prognosis explained by the treating physician	48 (40.33)
Not willing for surgery	4 (3.36)
Further treatment not available here and unable to transfer to the hospital being referred	4 (3.36)
**Others**	
No guarantee on the outcome of the baby	10 (8.40)
Cause of LAMA not stated	2 (1.68)

† **Note:** Some patients can have more than one reason for LAMA.

## DISCUSSION

In this study, out of 1352 total admissions in the NICU during the study period, the prevalence of LAMA was found to be 119 (8.80%). It is lower than the prevalence reported by similar studies from Nepal and India (15.6-25.4%).^5,7-9^ However other studies from various NICUs in Nepal have reported the prevalence of LAMA between 2.2-9.7%.^10,11^ This variability may be because of the differences in local socio-cultural factors, level of care provided by the centres and different timings of the study. A similar study from the NICU of Saudi Arabia however has reported the prevalence of Lama as 1.6%.^12^ It may be because of a better health system and no financial burden to the parents in their setup.

There was male predominance in this study 70 (58.82%) which is consistent with similar other studies from Nepal, India and Saudi Arabia (55-65%).^5,7,12^ Majority of the patients leaving against medical advice belonged to the disadvantaged ethnic groups like Janajati from Hill and Terai 73 (61.33%) and Dalits 15 (12.60%). This finding is consistent with a study from the NICU of Nepal.^7^ Similar studies from Pakistan and Nigeria have also reported low socioeconomic status among 72.1-91% of their patients who were self-discharged from their newborn care units.^13,14^

In this study, 60 (50.42%) of the patients took LAMA discharge within 2-7 days of admission which is consistent with the study from Nepal, India and Nigeria which also reported a majority of them leaving within 7 days (66-71.6%).^6,7,14^ However study from Saudi Arabia reported an average stay of 1 0 days before LAM A which is higher than our study (6.9 days).^12^ Almost one-fourth (24.3%) of our patients decided to LAMA within one day of admission which is consistent with similar other studies (20.7-34%).^6,7,15^

Seventy-two (60.50%) of the neonates taking LAMA were preterm which is consistent with the study from Nigeria which also reported prematurity (40%) as the commonest morbidity among their patients who were self-discharged.^14^ However, one study from India showed that term babies (62%) and babies with normal birth weight (59.8%%) were more prone to LAMA.^6^ This may be because term babies are relatively active unless very sick which gives a false impression to the parents that the neonate is well to take such a decision. Most other studies have reported sepsis (34-51.2%) as the most common morbidity among their LAMA patients.^5-7,15^ Birth asphyxia, respiratory distress syndrome of newborn (RDS), Neonatal jaundice and meconium aspiration syndrome (MAS) were among other major diagnoses in patients who took LAMA discharges which is corroborated by other studies.^5,7,13,15^

The most common reason for LAMA in our study was poor prognosis explained by the treating physician 48 (40.33%) followed by no improvement 40 (33.61%) which is consistent with the study from Pakistan who reported perceived poor clinical outcome 36.70% as the most common cause of LAMA.^13^ Lack of funds/unaffordability reported by 26.9% of parents of our patients was the most common cause for LAMA in other studies from Nepal, India and Nigeria (34.5-70%).^5,7,14,15^ This difference might be because most of the neonatal services in our NICU are provided free of cost to the patients through the free newborn care program of the Government of Nepal (GoN) reducing out-of-the-pocket expenditure. Still, a significant number of them taking LAMA due to financial reasons warrants revision of the program and inclusion of indirect financial burden of the family. A study from India however has reported wanting to take to a higher centre (25.15%) as the commonest cause for LAMA.^6^ These differences in reasons for LAMA may be attributable to the local socio-cultural factors, education and understanding of the parents, level of care provided by the hospitals and the cost of treatment imposed on the family. No one to take care of the farming/agriculture at home 24 (20.16%), forced to take LAMA by grandparents/relatives 14 (11.76%), perceived improvement 13 (10.92%), and no guarantee of the outcome of the baby 10 (8.40) were among the other important causes to take LAMA decision in our study. These reasons which have also been reported by other similar studies are real and important and thus cannot be ignored.^5-7,13,15^

Our study was based on data from the NICU of a single hospital with a small sample size conducted within a limited time period. Therefore, it cannot be generalised to the broader prospect. Furthermore, since this is a descriptive study, we were unable to find associations between the variables and LAMA discharges.

## CONCLUSIONS

The prevalence of leave against medical advice (LAMA) from the neonatal intensive care unit was lower than in other studies done in similar settings.
